# Shifts in Older Adults’ Social Support during Early Months of the COVID-19 Pandemic

**DOI:** 10.1093/geroni/igaa057.3498

**Published:** 2020-12-16

**Authors:** Heather Fuller, Andrea Huseth-Zosel, Emily Sturn, Shawn Carlson, Brittany Hofmann, Emily Kinkade, Bryce Van Vleet

**Affiliations:** North Dakota State University, Fargo, North Dakota, United States

## Abstract

In recent months, older adults have faced great health risks due to COVID-19, yet social distancing measures may also heighten risks to their social well-being. This mixed methods study explores changes in older adults’ social support satisfaction and interpersonal connections across the first few months of the COVID-19 pandemic. A Midwestern sample of 70 older adults aged 70-97 completed two phone interviews (April and June 2020) about their experiences with social distancing due to COVID-19. At both timepoints participants rated their satisfaction with social support and responded to open-ended questions about their interpersonal interactions, communication, and support in current daily life. Mean social support satisfaction significantly increased between the two interviews. Ensuing analysis of qualitative responses suggested this shift could reflect psychological adjustment to the circumstances or adaptation in methods of interpersonal connection over time. Emergent themes included: 1) increased family support and strain, 2) adaptable and flexible friendships, 3) isolation fatigue, and 4) communication through technology. Evaluation of change over time indicated divergent and shifting perceptions of social support as the pandemic endures. Findings suggest nuanced and diverse social support experiences amongst older adults, yet general patterns of initial shock to social support systems that subsided or adapted over time. Future research should build upon these findings to better understand older adults’ social support needs and seek to explore ways to best foster social connections during instances of forced social isolation or societal crises such as the current and ongoing global COVID-19 pandemic.

